# Clinical depiction, treatment and long term follow up characterization of a group of enthesitis related arthritis - juvenile idiopathic arthritis patients from a spanish pediatric tertiary hospital

**DOI:** 10.1186/1546-0096-12-S1-P181

**Published:** 2014-09-17

**Authors:** Samuel Hernandez Baldizon, Vicente Torrente-Segarra, Estibaliz Iglesias, Rosa Bou, Judith Sanchez Manubens, Joan Calzada Hernandez, Lilian López Núñez, Clara Giménez- Roca, Silvia Ricart, Joan Nolla Solé, Jordi Anton Lopez

**Affiliations:** 1 Pediatric Rheumatology Unit, Pediatrics Department, Hospital Sant Joan de Deu, Barcelona, Spain; 2 Department of Rheumatology, Hospital del Mar, Barcelona, Spain; 3 Department of Rheumatology, Hospital de Bellvitge, Barcelona, Spain

## Introduction

Enthesitis related arthritis (ERA) is a subtype of Juvenile Idiopathic Arthritis (JIA) that affects children >6 years of age. It presents with enthesitis, uveitis, peripheral and axial arthritis. It´s one of the less frequent subtypes of JIA. Data is scarse involving follow up and outcomes.

## Objectives

Describe a case series of JIA-ERA patients of a Spanish cohort in a tertiary pediatric hospital. Report response to oral (OR) and subcutaneous (SC) methotrexate (MTX) in insufficient/non-responders to NSAID/intra-articular steroid infiltrations in these patients. Relate the persistence of symptoms on follow-up and course of treatment.

## Methods

Retrospective and observational.Inclusion criteria: Edmonton classification criteria for ERA-JIA. Variables: age, sex, HLA-B27, date and delay of diagnosis, onset of symptoms, classification, start/finish of date of OR MTX, start/finish date of MTX SC, date of start of biologic treatment (as a variable of MTX inefficacy). Articular activity: number of swollen joints (NSJ) and number of painful joints (NPJ). Prospective data will be collected starting January 2014 of all ERA-JIA patients visited in our Unit. Local ethics committee approval was acquired.

## Results

11 patients all of which were male (100%), mean age of 15.3 (SD 4.6), age of first symptom 11.2, (SD 2.4), age of diagnosis 11.6 years (SD 2.3), delay in diagnosis 4.7 months (SD 6.3), follow up 55.6 months (SD 42.2). 90% were HLA-B27positive. The rest of the clinical data are summed up on table 1. Number of painful joints (NPJ) varied from 0-4, number of swollen joints (NSJ) between 0 and 8. 90.9% (10 patients) required MTX. Six received OR MTX (54.5%), 4 of these patients switched to SC MTX (66.7%) due to inefficacy. A total of 8 of 11 patients received SC MTX. The average dose of MTX was 14.6 mg/week (SD 3.9). Two (20%) patients continued MTX on transition to the adult rheumatology clinic. The average time on MTX was 13.1 months (SD 7.6). One patient suspended treatment with MTX because of digestive intolerance and another because of inefficacy. One patient (9%) started Etanercept because of partial response to both OR and SCMTX.

**Table 1:** 

Joint activity	NPJ at onset	NPJ at end of follow up	NSJ at onset	NSJ at end of follow up
Average	1	0	2.82	0.13

Standard deviation	1.34	0.00	3.28	0.35

## Conclusion

Patients had a mean age of 11 years at time of diagnosis, are male, HLA-B27 positive and have low level joint activity. The majority of patients that started OR MTX switched to SC MTX due to inefficacy or insufficient response, with a mean weekly dose of 15mg/weekSC MTX. The rate of side effects was low (<10%). Several patients required active treatment (DMARD/biologic) after 4 years of follow-up. Our findings should be taken with a grain of salt as this analysis is preliminary and we will be completing data from all of our JIA-ERA patients in coming months.

## Disclosure of interest

None declared.

